# The relationship between social support and learning engagement among Chinese preschool teacher trainees: the chain mediating effect of psychological capital and professional identity

**DOI:** 10.3389/fpsyg.2025.1687253

**Published:** 2025-10-27

**Authors:** Meng Zhang, Guanghua Wang, Li Li

**Affiliations:** ^1^College of Teacher Education, East China Normal University, Shanghai, China; ^2^College of Education, Shanghai Normal University Tianhua College, Shanghai, China; ^3^International Collaborative Programs and Services, Shanghai Normal University Tianhua College, Shanghai, China

**Keywords:** preschool teacher trainees, social support, learning engagement, psychological capital, professional identity

## Abstract

**Introduction:**

The development of the teaching workforce serves as a cornerstone of China’s strategy to become an educational power. In the context of demographic structural transformation, the cultivation of preschool education teachers faces new demands posed by the contemporary era. Investigating the learning engagement of preschool teacher trainees and exploring the influencing factors and their mechanisms of action are of significant importance for enhancing the learning quality of these students.

**Methods:**

Data were collected from 1,160 preschool teacher trainees in Shanghai, China, via four scales: social support scale, the psychological capital scale, the professional identity scale, and the learning engagement scale. This study employed SPSS and Model 6 of the SPSS macro program PROCESS 4.0 to analysis and test.

**Results:**

Social support is positively associated with learning engagement among preschool teacher trainees (*β* = 0.611, *p* < 0.001). Psychological capital can play a mediating role between social support and the learning engagement of preschool teacher trainees, with the mediating effect accounting for 36.99%. Professional identity can play a mediating role between social support and the learning engagement of preschool teacher trainees, with the mediating effect accounting for 11.78%. Social support can influence learning engagement through the chain mediating pathway of “psychological capital → professional identity,” with the mediating effect accounting for 31.59%.

**Discussion:**

Findings underscore the protective role of social support, psychological capital, and professional identity in academic contexts, suggesting that it promotes the learning engagement among preschool teacher trainees. Accordingly, this study proposes a tripartite educational model encompassing “external support—psychological empowerment—identity construction” to optimize the practices of talent cultivation for preschool teacher trainees.

## 1 Introduction

Teacher workforce development constitutes the foundational pillar of China’s national strategy for building a world-leading education system (Lu, 2023). As the bedrock of the national education system, the quality of preschool education teacher training not only determines the success of the sector’s transition from “scale expansion” to “connotation-based development,” but also exerts a direct impact on the sustainable development of high-quality preschool education in China. At present, China’s preschool teacher-preparation system is undergoing a tripartite transformation. Firstly, the implementation of the “Early Childhood Education Law” in 2025 will urge an improvement in the quality of teacher training. Secondly, the development of new qualitative productive forces has led to the innovation of teacher training models (Cheng, 2024). Thirdly, the onset of an era marked by persistently low fertility has precipitated structural shifts in the demand for preschool education teachers. Population-projection studies indicate that the number of children enrolled in kindergartens will decline continuously between 2023 and 2035, generating a potential phase-specific surplus of full-time preschool teachers (Hai and Gao, 2023). This trend has led to a “quality improvement paradox” in the development of preschool education programs: On the one hand, the standards for talent cultivation are continuously raised under policy-driven forces; on the other hand, the shrinking job market has intensified the weakening of the learning motivation of preschool teacher trainees and the crisis of professional identity among them (Cui and Zhang, 2023). This contradiction highlights the adaptability dilemma of the traditional training model. Therefore, how to maintain the learning engagement of preschool teacher trainees through the interaction of external support and internal driving mechanisms becomes the key to resolving the current predicament.

Learning engagement, as a key indicator for evaluating the quality of higher education talent cultivation, has gradually become the focus of educational psychology research in China and has received extensive attention. Learning engagement represents an extension of the concept of work engagement into the educational domain. It refers to an energetic and positive psychological state exhibited by students, encompassing vitality, dedication, and concentration (Schaufeli et al., 2002; Fang et al., 2008; Ni and Wu, 2011). It refers to a dynamic emotional state and positive mental state exhibited by students. Previous studies have confirmed that students’ learning engagement can positively predict their academic achievements (Wen et al., 2010), and it can also affect students’ learning satisfaction and learning experience, etc. (Wang and Wang, 2021), and even influences the formation and cultivation of the sense of professional mission among preschool teacher trainees (Chen et al., 2016). Thus, learning engagement plays a pivotal role in both students’ academic advancement and their subsequent career development (Tam, 2002). Furthermore, because individual development and growth are inseparable from the various contexts in which they exist (Bronfenbrenner, 1979), the formation mechanism of learning engagement can be explained from the perspective of the ecosystem theory. An empirical study on the influencing factors of students’ learning engagement has found that the interaction between individual internal factors and external environmental factors can enhance learning engagement (Wang and Wang, 2017). Further analysis reveals that the personal factors influencing learning engagement include learning motivation (Bian, 2024), self-efficacy (Zhou, 2023), and academic emotions (Guo, 2024), etc., while the environmental factors include teacher support (Liu, 2023), peer feedback (Liu, 2024), and family environment (Chang, 2024), etc.

Although current research has paid attention to the significant value and influencing factors of learning engagement, there is still a lack of sufficient studies on this particular group of preschool teacher trainees. In particular, there is a lack of systematic examination of the mechanism of the effects of these influencing factors. From the perspective of Bronfenbrenner’s (1979) ecosystem theory, the learning engagement of preschool teacher trainees is essentially the result of the interaction between the external environment and personal traits. This multi-level nested structure has not been fully revealed. Therefore, under the dual circumstances of current educational reform and changes in the employment situation, this study intends to deeply explore the internal and external factors influencing the learning engagement of preschool teacher trainees and their underlying mechanisms. Subsequently, it will propose targeted educational suggestions to provide scientific basis and practical guidance for improving the quality of talent cultivation.

### 1.1 Social support and learning engagement

Social support theory posits that the material and psychological resources individuals derive from their social networks can effectively modulate the dynamic equilibrium between stress and physical-mental wellbeing (Liu and Huang, 2010). Within this theoretical framework, the social support network comprising multiple entities such as family, schools, and communities plays a pivotal role in fostering individuals’ psychological security and enhancing their capacity for resource acquisition. Generally speaking, social support can be classified into two major categories in terms of nature: objective support and subjective support. The former is manifested in explicit forms of support such as material assistance and group participation, while the latter is reflected in implicit support elements such as the emotional experiences of being respected, understood, and cared for (Xiao, 1994). It is noteworthy that the efficacy realization of both objective and subjective support necessitates an individual’s cognitive acceptance and transformation process of such support. Ecosystem theory further posits that the social environment, as a critical component of the microsystem, serves as a fundamental supportive factor influencing individual learning behaviors and mental health (Yu et al., 2018). This perspective provides robust theoretical underpinnings for examining the mechanisms through which social support operates.

Within the domain of educational psychology, learning engagement is recognized as a relatively positive psychological learning disposition, easily influenced by the positive effects of diverse social support from sources such as schools and families. Empirical research indicates that school-based teacher autonomy support exerts a significantly positive predictive effect on middle-school students’ learning engagement; it satisfies their basic psychological needs for competence and relatedness (Chen et al., 2015), thereby generating sustained motivational outcomes. A recent study targeting university students majoring in preschool childhood education has demonstrated that teacher support and guidance serve as a crucial external support resource, effectively fostering students’ self-regulated learning, stimulating their intrinsic motivation, and consequently influencing their learning engagement in preschool education studies (Liu and Wang, 2024). The family support system also plays a pivotal role in the formation of learning engagement. A study on primary school students’ learning engagement reveals that families can provide material resources, academic guidance, and stress alleviation support, which may collectively foster students’ learning engagement through positive psychological and behavioral cycles (Wei and Dai, 2013). It is noteworthy that a higher socioeconomic status of families can provide greater social support, thereby fostering a more positive attitude toward learning and reinforcing individuals’ confidence in their learning engagement (Cheng, 2016). A substantial body of domestic and international research has further corroborated the universality of this relationship. A substantial body of both domestic and international research has further confirmed the universality of this relationship: the greater the perceived social support among students, the higher their level of learning engagement (Wang and Eccles, 2012). Research has substantiated that social support can enhance learning engagement among preschool teacher trainees (Zhang, 2015; Yin, 2021). Based on these findings, this study proposes the hypothesis:

*H1*: Social support will be positively associated with learning engagement among preschool teacher trainees.

### 1.2 The mediating role of psychological capital

According to the conservation of resources theory (COR), individuals obtain, maintain and utilize resources to cope with environmental challenges (Hobfoll, 1989). Psychological capital, as a core individual characteristic resource (Hobfoll, 2002), comprises four dimensions: self-efficacy, hope, resilience, and optimism (Luthans et al., 2007a). It can be seen that the COR can be used to explain the mechanism of psychological capital. That is, when an individual has sufficient psychological capital, they can effectively resist external pressure, maintain resource circulation, and achieve resource appreciation through positive coping strategies (Halbesleben et al., 2014). When applied to the student group, psychological capital can help college students face the pressure and setbacks in their studies with optimism, and engage in the learning process with a healthier and more positive mental state. Relevant research has confirmed that the psychological capital of student groups can positively predict learning engagement (Ding, 2015), and students with high levels of psychological capital also have a high degree of learning engagement (Xiang, 2024). Further analysis reveals that psychological capital has a synergistic effect on college students’ learning engagement, and its overall impact is stronger than the sum of its individual components (i.e., self-efficacy, hope, optimism, and resilience) (Mou and Huang, 2025).

Social support theory posits that regardless of an individual’s current level of social support, any increase in such support will lead to improved mental health outcomes (Chen et al., 2020). This demonstrates the pivotal role of social support as a supportive resource in maintaining and enhancing emotional wellbeing (Liu and Huang, 2010). A study on the relationship between social support and psychological capital demonstrates that social support constitutes one of the pivotal external variables influencing individuals’ positive psychological wellbeing (Zhu and Tao, 2022). Relevant research has also confirmed that individuals with a high level of social support possess more positive psychological capital, and social support has a significant positive influence on psychological capital (You et al., 2024). Drawing on the COR framework, social support—an external resource—exhibits a complementary relationship with psychological capital, an internal resource. When individuals perceive adequate social support, they are more confident in investing psychological resources into the field of learning. Consequently, as an external resource, social support can complement and augment the internal resource of psychological capital (Hobfoll, 1989), which in turn further enhances learning engagement (Xiang, 2024). Based on this theoretical framework, this study proposes the hypothesis:

*H2*: Preschool teacher trainees’ psychological capital mediates the relationship between social support and learning engagement.

### 1.3 The mediating role of professional identity

Professional identity is a crucial component of an individual’s self-identity (Salling, 2001). It refers to the emotional acceptance and recognition that learners develop following cognitive understanding of the disciplinary foundation of their chosen major, accompanied by positive external behaviors and a sense of internal appropriateness. It represents a process of integration encompassing emotional, attitudinal, and cognitive dimensions (Qin, 2009). In general, professional identity not only manifests as students’ positive affective attachment and vocational commitment to their major (Yang and Long, 2009), but also reflects their self-assessment of compatibility with the major based on professional cognition (Yu et al., 2021). Existing research has demonstrated that college students’ professional identity can significantly and positively predict their learning engagement (Chen and Wu, 2022; Zhang and Li, 2018). Focusing on the cohort of preschool teacher trainees as the research subjects, empirical studies have substantiated that a higher level of professional identity correlates with greater academic engagement (Wang, 2023; Zhang et al., 2020). As identity theory posits, the degree to which individuals identify with a particular identity or social role influences their behavioral manifestations (Stryker and Burke, 2000). Preschool teacher trainees who demonstrate strong professional identity alignment are more likely to exhibit goal-congruent behaviors, such as proactive learning and deep engagement.

Multiple empirical studies on preschool teacher trainees have demonstrated a positive correlation between professional identity and social support (Chen, 2022; Hu, 2022), with the latter exerting a direct influence on the former (Chen et al., 2023). An empirical study on preschool teacher trainees reveals that multifaceted support from significant others, including relatives, peers, instructors, young children, and kindergarten principals, exerts influence on teacher trainees’ professional identity formation through its impact on their professional aspirations (Ma, 2024). According to Self-Determination Theory (SDT), social support enhances intrinsic motivation by satisfying individuals’ psychological needs for autonomy, competence, and relatedness, which in turn facilitates positive behavioral outcomes, such as increased engagement in learning (Deci and Ryan, 1985). In other words, support from teachers, classmates, and families can enhance students’ professional identity. Furthermore, a higher level of professional identity contributes to increased learning motivation and enhanced learning engagement. It is worth noting that a study on special education for teacher trainees has found that professional identity mediates the relationship between classroom climate and learning engagement, indicating that psychological-cognitive factors (e.g., professional identity) can act as a bridge between environmental factors (e.g., social support) and learning engagement (Chen and Wu, 2022). Therefore, when students perceive the social support around them, their professional identity (i.e., the internalization of the value of their major) will be strengthened, thereby enhancing their level of learning engagement. In summary, this study proposes the hypothesis:

*H3*: The professional identity of preschool teacher trainees plays a mediating role in the relationship between social support and learning engagement.

### 1.4 The chain mediating effect of psychological capital and professional identity

Previous research has separately demonstrated the relationships between each pair of social support, learning engagement, psychological capital, and professional identity. Research has substantiated a significant positive correlation between professional identity and psychological capital among university students (Chen et al., 2016). In-depth analysis reveals that all four dimensions of psychological capital—self-efficacy, hope, optimism, and resilience—exhibit significant positive associations with the formation of professional identity and play an active role in its development (Hu, 2022). A high level of psychological capital facilitates college students in establishing positive perceptions of their major, forming emotional connections with the discipline, recognizing its professional value, and translating such recognition into concrete actions, thereby achieving strong professional identity (Xu et al., 2023). Furthermore, students with higher levels of psychological capital exhibit a greater propensity for optimistic assessment of their majors’ future development prospects, thereby enhancing their professional identity (Sun et al., 2020). Empirical studies have corroborated that all four dimensions of psychological capital positively predict college students’ professional identity, with the hope dimension demonstrating particularly pronounced effects (Chen et al., 2016). An empirical study on vocational education teacher candidates has demonstrated that psychological capital exerts a significantly positive influence on professional identity, and the enhancement of psychological capital contributes to strengthening teacher candidates’ professional identity (Wang and Yuan, 2024). Consequently, building upon the research hypotheses H1, H2, and H3 previously proposed, this study advances this hypothesis:

*H4*: Psychological capital and professional identity function as chain mediators in the relationship between social support and learning engagement.

**Figure 1:**
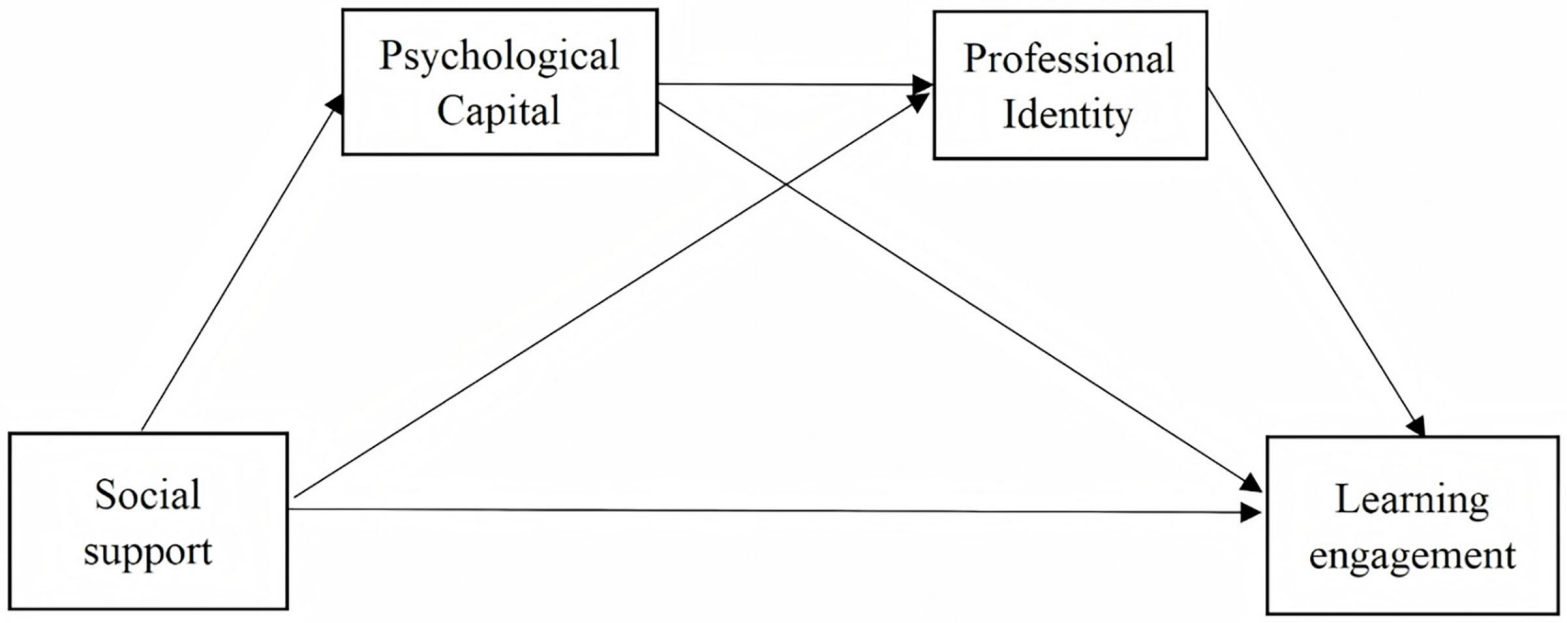
Hypothesized model.

In summary, from the perspective of ecosystem theory, social support, psychological capital, and professional identity constitute significant factors influencing college students’ learning engagement, and potential interaction effects among these variables may exist. Previous studies have also demonstrated significant pairwise correlations among social support, professional identity, psychological capital, and learning engagement. However, the interactive mechanisms among these multiple factors remain unclear, and research specifically targeting preschool teacher trainees is particularly limited. Therefore, this study adopts a multi-perspective theoretical framework encompassing Social Support Theory, COR Theory, and Identity Theory to investigate the interrelationships among social support, psychological capital, professional identity, and learning engagement. It establishes a chain mediation model of “social support → psychological capital → professional identity → learning engagement,” with the objective of elucidating the multi-level mechanisms through which external support systems are transformed into learning engagement. The proposed model is illustrated in Figure 1.

## 2 Materials and methods

### 2.1 Research participants

This study employed a convenience sampling method to recruit 1,195 undergraduate students majoring in preschool education from four Applied-oriented undergraduate institutions in Shanghai for participation in the survey. After screening the collected data, 35 questionnaires were excluded due to incomplete responses, resulting in a final analysis of 1,160 college students with a validity rate of 97.07%. The sample comprised 204 freshmen (17.586% of the total), 180 sophomores (15.517%), 318 juniors (27.414%), and 458 seniors (39.483%). Among the three categories of major selection preferences, 679 students (58.534% of the total) chose their majors independently, 450 students (38.793%) followed parental or third-party guidance, and 31 students (2.672%) were enrolled through program adjustment pathways. The sample comprised 49 male participants (4.224% of the total) and 1,111 female participants (95.776% of the total).

### 2.2 Measures

#### 2.2.1 Social support scale

This study utilized the Social Support Rating Scale developed by Xiao (1994) for measurement purposes. The scale comprises three dimensions: subjective social support, objective social support, and utilization of social support, with a total of 10 items. Among them, subjective social support (e.g., “My parents fully support me”) reflects an individual’s emotional experience and perceived satisfaction of being supported, understood, and respected within their social environment; objective social support (e.g., “I have received financial assistance or practical problem-solving help from family, friends, or others during emergencies”) pertains to the tangible aid and support derived from social networks and group relationships; while the utilization of social support (e.g., “When facing difficulties, I frequently seek help from parents, relatives, friends, or organizations”) demonstrates the individual’s cognitive assimilation and transformation process regarding various forms of support. The scale utilizes a 4-point Likert-type scoring system, where higher scores reflect a stronger perception of social support among students. In this study, the scale achieved a Cronbach’s *α* coefficient of 0.749, with internal consistency of each item exceeding 0.7, suggesting satisfactory reliability.

#### 2.2.2 Learning engagement scale

This study utilizes the Utrecht Work Engagement Scale-Student (UWES-S), originally developed by Schaufeli et al. (2002) and later translated and adapted by Chinese scholars Fang et al. (2008). The scale consists of three dimensions—vitality, dedication, and concentration—comprising a total of 17 items. Vitality (e.g., “I can sustain prolonged learning sessions without requiring breaks”) refers to the degree of energy investment and proactive engagement exhibited during the learning process, with higher scores indicating a higher level of learning vitality. Dedication (e.g., “I find the learning objectives clear and highly meaningful”) refers to the positive emotional experiences associated with the learning process, with higher scores indicating a higher degree of positive affect experienced during learning. Concentration (e.g., “Time seems to pass quickly when I study”) refers to the extent of focused attention maintained during the learning process, with higher scores indicating a higher level of mental engagement in learning activities. The scale employs a positive scoring system ranging from 1 to 7, where 1 denotes “never” and 7 signifies “always/daily” in terms of frequency of occurrence. Higher scores indicate greater levels of learning engagement. The Cronbach’s alpha coefficient of this scale in the present study was 0.972, with all items demonstrating internal consistency exceeding 0.93, indicating high reliability.

#### 2.2.3 Psychological capital scale

This study utilizes the Psychological Capital Scale developed by Wang et al. (2011). This instrument, which was adapted by Wang and colleagues from Luthans’ original scale through academic revision, classifies hope and optimism within the same dimension to better align with the characteristics of the Chinese university students. The scale consists of four core dimensions and comprise a total of 16 items. These dimensions include: self-confidence (e.g., “In team activities, I believe I can fulfill my expected role”), resilience (e.g., “I can always recover quickly when encountering setbacks”), optimism (e.g., “When facing difficulties, I firmly believe that ‘sunshine comes after the storm’“), and responsibility (e.g., “I strive to fulfill commitments I have made”). This scale utilizes a 5-point Likert-type scoring system ranging from 1 to 5, where 1 corresponds to “strongly disagree” and 5 corresponds to “strongly agree,” with higher scores reflecting higher levels of psychological capital. The internal consistency of this scale in the present study demonstrated a Cronbach’s *α* coefficient of 0.941, with item-level consistency exceeding 0.93, indicating high reliability.

#### 2.2.4 College student professional identity scale

This study utilizes the College Student Professional Identity Scale, originally developed by Qin (2009), as the primary measurement instrument. The scale consists of four distinct dimensions: cognition, affection, behavior, and suitability, encompassing a total of 23 items. The cognitive dimension (e.g., “Overall, I have a comprehensive understanding of my major”) reflects the level of familiarity with the specialized field, where higher scores indicate greater mastery of the subject matter. The affective dimension (e.g., “I am highly confident about the career prospects of my major”) refers to the degree of emotional preference for an academic discipline, with higher scores indicating more favorable affective attitudes toward the major. The behavioral dimension (e.g., “I actively participate in professional practice activities related to my major”) refers to the manifestation of behavior within the academic discipline, with higher scores indicating greater proactivity in professional conduct. The suitability (e.g., “My major aligns with my strengths”) refers to the extent of congruence between an individual’s major and their personal aptitudes, where higher scores indicate a stronger alignment. The scale employs a positive scoring system ranging from 1 to 5, where 1 indicates “strongly disagree” and 5 signifies “strongly agree,” with higher scores reflecting greater professional identity. The Cronbach’s alpha coefficient of the scale in this study was 0.960, with all items demonstrating internal consistency exceeding 0.95, indicating high reliability.

### 2.3 Data collection and processing

Initially, the Institutional Review Board (IRB) at the researchers’ university conducted a comprehensive review and granted formal approval for this study. The official approval number is (2024) No. 01. Additionally, the study was carried out with informed consent obtained from all participants, with professional instructors from four academic institutions administering the survey and delivering standardized instructions via the specialized online platform “Wenjuanxing.” Subsequently, this study employed SPSS 23.0 to conduct the common method bias test on the collected valid data, along with descriptive statistics and correlation analyses of multiple variables. A chain mediating effect was established using Model 6 of the SPSS macro program PROCESS 4.0, with the bootstrap method employing bias-corrected percentile (5,000 resamples at 95% confidence interval) to examine this chain mediating effect.

## 3 Research results

### 3.1 Common method bias test

This study primarily utilized questionnaires for data collection, which relied on student self-assessment, potentially leading to common method bias. To mitigate this concern, Harman’s single-factor test (Matthews et al., 2006) was implemented to conduct the common method bias test. The results indicate that an exploratory factor analysis of the data without rotation yielded 14 factors with eigenvalues exceeding 1. The first principal component explained 28.485% of the total variance, which is substantially below the critical threshold of 40%. Therefore, findings of this study demonstrate the absence of significant common method bias.

### 3.2 Descriptive and correlational analyses of study variables

To elucidate the underlying relationships among the variables, descriptive statistics and correlation analyses were performed on the key variables related to preschool teacher trainees, with the results summarized in Table 1. The results indicate a statistically significant negative correlation between gender and social support (*r* = −0.067, *p* < 0.05), while academic year demonstrates a positive correlation with learning engagement (*t* = 0.083, *p* < 0.01). Notably, major choice exhibits significant negative correlations with multiple variables: social support (*t* = −0.129, *p* < 0.001), psychological capital (*t* = −0.135, *p* < 0.001), professional identity (*t* = −0.255, *p* < 0.001), and learning engagement (*t* = −0.104, *p* < 0.001). It is therefore apparent that that gender, academic year, and major choice among preschool teacher trainees demonstrate a moderate level of correlation with the key research variables, thereby necessitating their inclusion as control variables in subsequent data analyses.

**Table 1:** Descriptive statistics and correlation analysis of variables.

Variables	Mean	Standard deviation	1	2	3	4	5	6	7
1. Gender	1.958	0.201	1						
2. Academic year	2.888	1.114	0.04	1					
3. Major choice	1.441	0.548	0.028	0.037	1				
4. Social support	2.767	0.462	−0.067*	−0.012	−0.129***	1			
5. Psychological capital	3.727	0.606	0.029	0.037	−0.135***	0.363***	1		
6. Professional identity	3.785	0.621	0.055	0.031	−0.255***	0.302***	0.624***	1	
7. Learning engagement	4.135	1.05	0.018	0.083**	−0.104***	0.275***	0.556***	0.598***	1

*n* = 1,160, *p < 0.05, **p < 0.01, ***p < 0.001.

As illustrated in Table 1, preschool teacher trainees scored above average on social support (*M* = 2.767), psychological capital (*M* = 3.727), professional identity (*M* = 3.785), and learning engagement (*M* = 4.135). Furthermore, statistically significant positive correlations (*p* < 0.001) were observed between each pairwise combination of these variables: social support, psychological capital, professional identity, and learning engagement. Given the statistically significant positive correlations identified among all pairs of the four research variables within the preschool teacher trainee sample, the data is considered suitable for further analysis using a chain mediation model.

### 3.3 The chain mediating effect test

This study constructed a chain mediating model with social support designated as the independent variable, learning engagement as the dependent variable, and gender, academic year, and major choice included as control variables, while psychological capital and professional identity functioned as mediating variables. The analysis was carried out using Model 6 of the SPSS macro PROCESS 4.0 (Hayes, 2013), with the findings summarized in Table 2.

**Table 2:** Regression analysis of variable relationships in the chain mediation model.

Variables	Learning engagement		Psychological capital		Professional identity		Learning engagement	
	*β*	*t*	*β*	*t*	*β*	*t*	*β*	*t*
Gender	0.179	1.22	0.161	1.958	0.148	2.137*	−0.07	−0.594
Academic year	0.083	3.132**	0.023	1.585	0.008	0.682	0.056	2.636**
Major choice	−0.142	−2.608**	−0.102	−3.371***	−0.192	−7.497***	0.086	1.935
Social support	0.611	9.481***	0.466	12.936***	0.102	3.153**	0.120	2.161*
Psychological capital					0.586	23.721***	0.485	9.454***
Professional identity							0.707	14.129***
*R^2^*	0.09		0.145		0.426		0.42	
Adjusted *R^2^*	0.086		0.142		0.424		0.417	
*F* value	28.437***		48.955***		171.578**		139.291***	

*n* = 1,160, *p < 0.05, **p < 0.01, ***p < 0.001.

**Figure 2:**
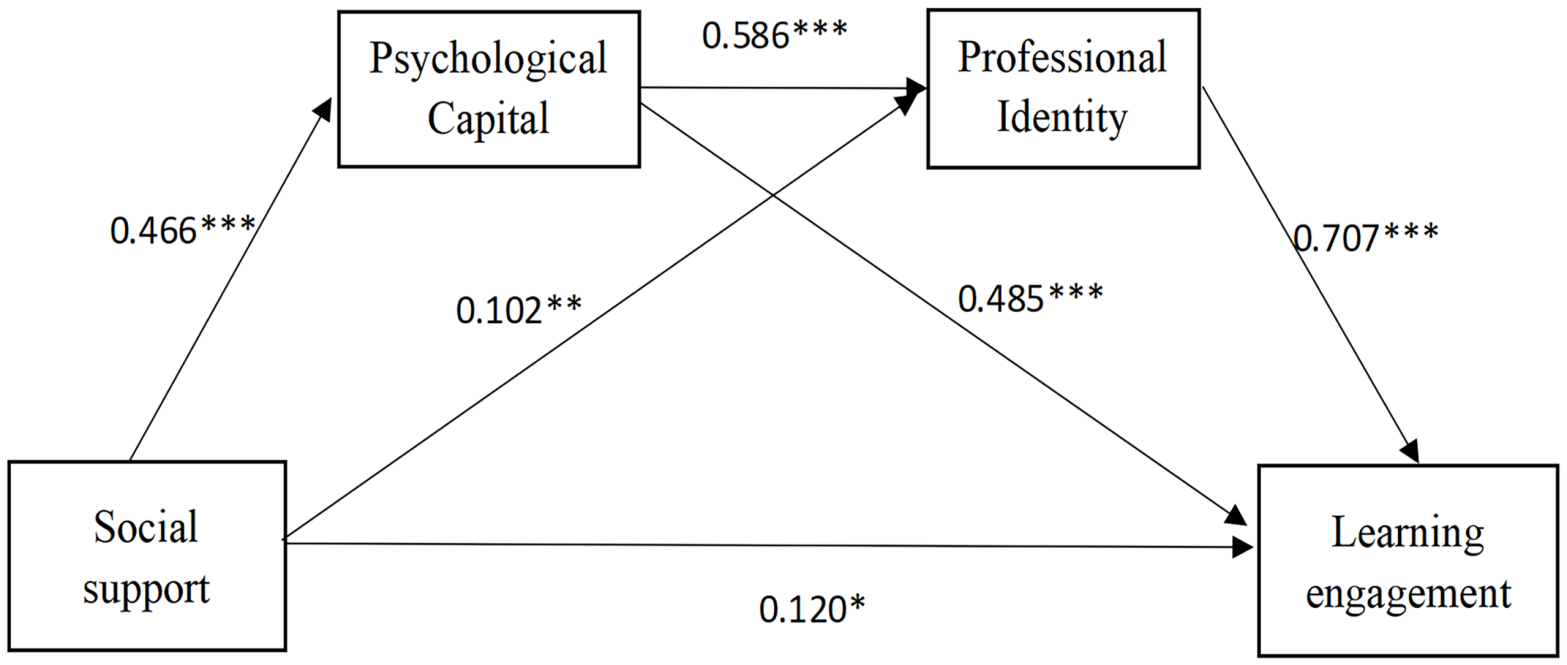
The chain mediation model of social support, psychological capital, professional identity, and learning engagement, *p < 0.05, **p < 0.01, ***p < 0.001.

As shown in Table 2, prior to the inclusion of the mediating variables, social support was found to be positively associated with learning engagement among preschool teacher trainees (*β* = 0.611, *p* < 0.001), This result confirms Hypothesis H1. Following the inclusion of the two mediating variables, psychological capital and professional identity, social support was found to be positively associated with psychological capital (*β* = 0.466, *p* < 0.001), and psychological capital was positively related to learning engagement (*β* = 0.485, *p* < 0.001). These findings indicate that psychological capital functions as a mediating variable in the relationship between social support and learning engagement, thereby providing empirical support for Hypothesis H2. Social support was found to be positively associated with professional identity (*β* = 0.102, *p* < 0.01), and professional identity, in turn, was found to be positively associated with learning engagement (*β* = 0.707, *p* < 0.001). These findings indicate that professional identity functions as a mediating variable in the relationship between social support and learning engagement, thereby offering empirical support for Hypothesis H3. Psychological capital was found to be positively associated with professional identity (*β* = 0.586, *p* < 0.001), suggesting that psychological capital and professional identity function as chain mediators in the relationship between social support and learning engagement, thereby providing empirical support for Hypothesis H4. Furthermore, after incorporating the mediating variables, social support continues to be positively associated with learning engagement (*β* = 0.120, *p* < 0.05). In conclusion, the hypothesized chain mediation model has been empirically validated, as illustrated in Figure 2.

To further explore the independent mediating roles of psychological capital and professional identity, as well as their chain mediating effect, the bias-corrected percentile Bootstrap method (5,000 resamples at 95% confidence interval) was employed, as shown in Table 3. The 95% confidence intervals for all identified mediating pathways did not include zero, thereby confirming the statistical significance of the mediating effects. The results indicate that, firstly, the indirect effect of the “social support → psychological capital → learning engagement” pathway is 0.226, with a confidence interval of (0.071, 0.134), which confirms a statistically significant mediating effect. This represents 36.99% of the total effect, suggesting that psychological capital functions as a mediating mechanism through which social support influences learning engagement. Secondly, the indirect effect of the “social support → professional identity → learning engagement” pathway was 0.072, with a confidence interval of (0.011, 0.054), indicating a statistically significant mediating effect that accounted for 11.78% of the total effect. This demonstrates that professional identity serves as a mediating factor in the relationship between social support and learning engagement. Thirdly, the indirect effect of the “social support → psychological capital → professional identity → learning engagement” pathway was 0.193, with a confidence interval of (0.066, 0.108), indicating a statistically significant mediating effect that accounted for 31.59% of the total effect. This demonstrates that psychological capital and professional identity serve as chain mediators in the relationship between social support and learning engagement. The direct effect was 0.120, while the total effect reached 0.611.

**Table 3:** Analysis of the significance test results for mediating effects.

Pathways	Effect (effect value)	Boot SE	Effect proportion	95% bootstrap CI
Social support ⇒ psychological capital ⇒ learning engagement	0.226	0.016	36.99%	(0.071, 0.134)
Social support ⇒ professional identity ⇒ learning engagement	0.072	0.011	11.78%	(0.011, 0.054)
Social support ⇒ Psychological capital ⇒ professional identity ⇒ learning engagement	0.193	0.011	31.59%	(0.066, 0.108)
Total mediating effect	0.491	0.02	80.36%	(0.178, 0.0.256)
Direct effect	0.120	0.055	19.64%	(0.011, 0.228)
Total effect	0.611	0.064	100.00%	(0.485, 0.737)

## 4 Discussion

This study focuses on preschool teacher trainees as research subjects, analyzing the relationship between social support and learning engagement while verifying both the independent mediating effects of psychological capital and professional identity, as well as their chain mediating mechanism. Amid the contraction of the teacher employment market caused by declining fertility rates, preschool teacher trainees are facing dual pressures of “rising demands for pedagogical training quality” and “intensified competition for teaching positions.” The waning learning engagement among these students has emerged as a prominent issue constraining the quality of talent cultivation, with preschool education majors being particularly affected. This study investigates the multilevel mechanism of social support’s impact on preschool teacher trainees’ learning engagement in the new era context, aiming to provide empirical evidence for optimizing their professional development and talent cultivation.

### 4.1 The relationship between social support and learning engagement among preschool teacher trainees

This study reveals a positive correlation between social support and the learning engagement of early childhood education majors, a finding that is consistent with prior research (Hou, 2020). Furthermore, this finding aligns with the “Main Effect Hypothesis” of social support theory. The main effect hypothesis posits that social support universally enhances individuals’ psychological well-being, irrespective of whether they are exposed to stressful circumstances (Cohen and Wills, 1985). Moreover, individuals with higher levels of social support are more likely to transform external attention and trust into intrinsic motivation, thereby adjusting cognitive processes and actively engaging in learning activities (Duckworth and Carlson, 2015), while demonstrating more resolute and vigorous learning engagement (Zhang, 2021).

In terms of objective support, higher education resource allocation (Lian, 2022) and family economic capital (Yang, 2024) can establish a stable learning foundation for preschool teacher trainees by improving learning environments and alleviating material constraints, thereby enhancing preschool teacher trainees’ learning engagement. As for subjective support, encouragement from family members, teachers, and peers conveys positive messages of understanding, recognition, and trust (Kong and You, 2013), thereby serving as an effective motivator for preschool teacher trainees’ learning engagement. It is noteworthy that positive peer relationships (Wang, 2013; Wang et al., 2024) and teachers’ emotional support contribute to the development of intrinsic learning motivation by enhancing students’ relatedness, self-efficacy, and interpersonal connectivity. This finding provides a theoretical foundation for establishing a “demand-oriented” preschool teacher training system, suggesting that educators need to systematically integrate resource-based support, relational support, and emotional support to facilitate the effective transformation of social support into learning engagement.

### 4.2 Psychological capital mediates the relationship between social support and learning engagement

This study confirms the mediating role of psychological capital between social support and learning engagement among preschool teacher trainees, a finding consistent with previous research (Song and Xu, 2022). This study identifies psychological capital as the mediating mechanism through which external support translates into learning engagement, whereby social support indirectly enhances preschool teacher trainees’ sustained engagement to professional learning by elevating their psychological capital levels. This finding provides a more profound analysis of the pathway through which social support operates from the perspective of the COR. The COR posits that optimal resource utility can only be achieved through the activation of an individual’s intrinsic resources (Hobfoll, 1989). Externally acquired resources (social support) can be transformed through individual competitive advantage resources—such as psychological capital (Luthans et al., 2007b)—into intrinsic psychological resilience, self-affirmation, and goal motivation, thereby enhancing learning engagement (Avey et al., 2011).

Generally speaking, preschool teacher trainees can access not only institutional resources, faculty mentorship, family support, and peer assistance as forms of social support, but also receive professional guidance and instruction from kindergarten principals and lead teachers during their practicum placements. This study integrates the learning environments and authentic contexts of preschool teacher trainees, conceptualizing social support as a critical external resource and psychological capital as an internal resource. It clarifies the underlying mechanism through which resource conversion facilitates the “resource gain spiral” effect (Hobfoll, 2011), thereby enriching theoretical perspectives in the field of teacher education. Simultaneously, this study validates the mediating role of psychological capital and empirically substantiates the resource conversion framework of “social support—psychological capital—learning engagement” through quantitative analysis. The findings highlight the importance of fostering psychological capital, establishing an empirical basis for refining the cultivation and professional development trajectories of preschool teacher trainees.

### 4.3 Professional identity mediates the relationship between social support and learning engagement

This study validates the mediating role of professional identity between social support and learning engagement among preschool teacher trainees, a finding consistent with prior research outcomes (Yu et al., 2024). This study provides empirical evidence that professional identity functions as a key psychological mechanism through which external support is translated into learning engagement, whereby social support can indirectly enhance the learning engagement of preschool teacher trainees by strengthening their professional identity. This finding can be interpreted through the theoretical framework of Self-Determination Theory (SDT). SDT posits that individual behavior and psychological growth are driven by three fundamental psychological needs: autonomy, competence, and relatedness (Deci and Ryan, 1985). In this study, social support facilitates the internalization of extrinsic professional goals into professional identity by addressing teacher trainees’ basic psychological needs, thereby stimulating their intrinsic learning motivation and ultimately promoting sustained engagement in learning behaviors (Ryan and Deci, 2000). This provides empirical evidence for the application of SDT in the educational domain, while also revealing that need satisfaction does not directly trigger behavioral engagement, but rather facilitates motivational transformation through professional identity, which serves as a higher-order cognitive integration process.

For instance, teachers’ in-class support can enhance students’ professional self-efficacy and promote their learning autonomy (Zhang, 2024). At the same time, it may also foster and strengthen students’ sense of belonging to their academic major or institution (Li, 2017). In addition, professional identity, as an internalized form of motivation, can encourage students to actively engage in the professional learning process voluntarily (Zhang and Li, 2018). Furthermore, supplementary analysis grounded in the COR reveals that social support, as a contextual resource, generates an augmentation effect of psychological resources through the cognitive restructuring of professional identity (Hobfoll, 1989). Based on the comprehensive analysis, it highlights the necessity for higher education institutions to prioritize the development of professional identity among teacher trainees by implementing a dual-mentorship system that integrates academic instructors with industry practitioners, as well as enhancing professional practicum experiences, thereby reinforcing their professional cognition and affective commitment.

### 4.4 Psychological capital and professional identity exert a chain mediating effect between social support and learning engagement

This study demonstrates that the chain mediation model composed of “psychological capital—professional identity” represents a key mechanism through which social support influences the learning engagement of preschool teacher trainees, thereby substantiating the operational mechanisms of the ecosystem. The findings empirically substantiate that preschool teacher trainees’ learning engagement is fundamentally a product of the interaction between external environmental factors and intrinsic personal traits, which aligns with the research findings from a study on special education teacher candidates (Chen and Wu, 2022). Further analysis indicates that social support contributes to the development of psychological capital among preschool teacher trainees, with the accumulation of this capital subsequently promoting the formation of professional identity. Ultimately, the intrinsic motivational function of professional identity enhances the level of learning engagement. This conclusion is consistent with the hypothesis of the “resource gain spiral” effect in the COR (Hobfoll, 2011).

Refer to the above analysis approach, social support is an external resource, psychological capital is an intrinsic resource. Preschool teacher trainees with a high degree of professional identity can effectively integrate and leverage both external and internal resources. By fulfilling individuals’ basic psychological needs, they develop internalized motivation (SDT) (Deci and Ryan, 2000), which directly influences the direction and persistence of learning behaviors, ultimately enhancing their level of learning engagement. In conclusion, this chain mediation model delineates a comprehensive pathway whereby external resources (social support) influence internal psychological resources (psychological capital), subsequently leading to the internalization of motivation (professional identity), and ultimately translating into learning engagement. Therefore, this study proposes a trinity education model based on the sequential mediating framework—“external support, psychological empowerment, and professional identity”—to enhance understanding of the professional development mechanism among preschool teacher trainees. Specifically, on the one hand, an external home-school collaborative education system can be established to support the professional learning of preschool teacher trainees. On the other hand, through multi-level intervention strategies—such as psychological capital enhancement programs and professional identity cultivation initiatives—the learning outcomes of preschool teacher trainees can be systematically improved.

## 5 Conclusions and recommendations

### 5.1 Conclusion

Based on an empirical analysis of the preschool teacher trainee cohort, this study yields the following conclusions. Firstly, a statistically significant positive correlation is observed among social support, psychological capital, professional identity, and learning engagement (*p* < 0.001). Secondly, Social support is positively associated with learning engagement among preschool teacher trainees. Thirdly, psychological capital and professional identity each serve as independent mediators in the relationship between social support and learning engagement. Fourthly, social support can influence learning engagement among preschool teacher trainees through the chain mediating pathway of “psychological capital → professional identity.” This indicates that social support, psychological capital, and professional identity are protective factors promoting students’ academic success.

### 5.2 Educational recommendations

Research findings indicate that social support, psychological capital, and professional identity represent key determinants influencing the learning engagement of preschool teacher trainees. This study integrates the theoretical foundations of social support, psychological capital, self-determination, and identity formation. Drawing upon the preceding theoretical discussion and empirical analysis, it proposes a tripartite educational model encompassing “external support—psychological empowerment—identity construction” to optimize the practices of talent cultivation for preschool teacher trainees, thereby fostering their learning engagement.

#### 5.2.1 Establish a collaborative home-school educational mechanism and refine a demand-driven social support system

Social support originating from family and educational institutions serves as a critical contextual factor that influences preschool teacher trainees’ learning engagement. To promote the holistic development of teacher trainees, the higher education institution should prioritize conducting comprehensive research and duly acknowledge the current academic and living needs of preschool education majors. Simultaneously, the provided social support should account for both explicit resource-based assistance and implicit emotional sustenance. Specifically, on the one hand, families can offer essential material support by providing teacher candidates with necessary academic resources such as textbooks and electronic devices. On the other hand, educational institutions can enhance professional support for preschool teacher trainees by revising training objectives, refining curriculum design, and offering experiential learning opportunities. Through coordinated collaboration between families and schools, a joint home-school communication platform should be established to construct a comprehensive, multi-tiered social support system, thereby effectively enhancing the learning motivation of preschool teacher trainees.

#### 5.2.2 Implement a tiered program for cultivating psychological capital to promote the development among preschool teacher trainees

In response to the prevalent career development anxieties arising from the current contraction of the job market and the widespread phenomenon of involution, higher education institutions should place significant emphasis on and actively develop a systematic psychological capital cultivation program. Particular attention must be given to incorporating a comprehensive consideration of individual differences in psychological capital levels among teacher trainees when designing this program, thereby establishing a tiered psychological capital cultivation framework. On the one hand, universities can. To cultivate students’ confident and optimistic attitudes through the systematic psychological capital cultivation program (Dou, 2024). On the other hand, through the implementation of challenging tasks at varying difficulty levels (e.g., professional skills competitions), the academic resilience of preschool teacher trainees can be systematically enhanced via targeted training interventions. Furthermore, higher education institutions should proactively foster collaboration between families and schools to jointly cultivate a sense of hope and an optimistic mindset among preschool teacher trainees.

#### 5.2.3 Fostering professional identity development communities to refine the practice-based educational cultivation system

The meticulous cultivation of preschool teacher trainees’ professional identity can be effectively realized through a dual-mentorship system comprising “university faculty and industry practitioners” (Chen and Zhang, 2019). This approach further enhances the practicum and internship components of professional training, thereby strengthening the practice-oriented education system for talent development. On the one hand, it is essential to implement immersive practical training programs. Freshmen shall participate in a job shadowing program, in which students are guided by dual mentors to conduct weekly observations of educational institutions’ operations to cultivate foundational professional awareness. During the sophomore year, micro-teaching practices can be implemented, incorporating modules such as collaborative educational case analysis and reflective teaching practicum sessions to promote the transformation of social support into professional identity. During their junior and senior years, teacher candidates should be guided to plan and implement educational teaching programs while fostering the development of professional cognition and affective commitment. On the other hand, utilizing big data capabilities facilitates the establishment of a preschool education resource repository, which provides students with comprehensive teaching plans, case study analysis in instructional practice, and narratives of professional development within the teaching profession. In addition, through activities such as distinguished-alumni sharing sessions and career-planning workshops, students’ psychological resource reserves are enhanced by deepening their understanding of professional prospects, thereby fostering greater enthusiasm for learning.

## 6 Research contributions and limitations

This study focuses on the cohort of preschool education majors, extending the frontiers of teacher education research through both theoretical and empirical approaches. On the theoretical front, this study, grounded in the theoretical framework of “how external support transforms into sustainable learning engagement,” proposes a dynamic adaptability perspective of social support. This study innovatively extends the COR from research on resource depletion to proactive pathways for the enhancement of psychological capital. Moreover, it conceptualizes professional identity as the mediating mechanism between need satisfaction and behavioral engagement in SDT, thereby expanding the explanatory scope of SDT within the domain of learning motivation transformation. At the practical level, this study proposes the establishment of a “demand-oriented” teacher training system through home-school collaboration. It highlights the necessity of synergistically integrating explicit resource guarantee with implicit relational support through strategies such as the dual-mentor mechanism, immersive practicum experiences, and resource database development. Furthermore, this study advocates for enhancing psychological capital cultivation and the internalization of professional identity, thereby offering systematic recommendations to optimize preschool teacher education by addressing both resource allocation mechanisms and motivational transformation pathways.

This study is subject to several limitations. Firstly, the sample is confined to preschool teacher trainees in Shanghai, which may limit the generalizability of the findings and calls for further empirical validation. Therefore, future research should aim to broaden both the geographical scope and the sample size. Secondly, this study primarily utilizes a cross-sectional research design with data collected at a single time point. In the future, longitudinal follow-up studies or cross-lagged panel designs could be employed to further investigate the causal relationships among variables in greater depth. Thirdly, although this study has confirmed the chain mediating effect of psychological capital and professional identity in the relationship between social support and learning engagement, it does not address more specific variable distinctions, such as a comparative analysis of subjective versus objective support within the construct of social support. In future research, additional variables may be incorporated to further examine the underlying mechanisms of the relationships among them.

## Data Availability

The raw data supporting the conclusions of this article will be made available by the authors, without undue reservation.
